# Center of Mass (CoM) Motions and Foot Placement During Treadmill Walking Using One Time-of-Flight Camera

**DOI:** 10.3390/s25185850

**Published:** 2025-09-19

**Authors:** Joshua T. Chang, Alisha Ragatz, Anjana Ganesh, Ana P. Quiros Padilla, Mikayla R. Devins, Christina V. Mihova, John G. Milton

**Affiliations:** 1 Department of Neurology, Dell Medical School, The University of Texas at Austin, Austin, TX 78712, USA; anjana.ganesh01@utrgv.edu (A.G.); aq3696@utexas.edu (A.P.Q.P.); mikayla.devins@austin.utexas.edu (M.R.D.); mihovaca@ucmail.uc.edu (C.V.M.); john.milton@austin.utexas.edu (J.G.M.); 2 Oden Institute of Computational Engineering and Sciences, The University of Texas at Austin, Austin, TX 78712, USA; alisha.ragatz@austin.utexas.edu

**Keywords:** gait analysis, time-of-flight camera, portable treadmill, CoM movements, foot placement

## Abstract

Assessing the fall risk of a patient in a busy clinical setting is challenging. Tests such as the timed-up-and-go test and narrow beam walking are difficult to perform due to space restrictions. Moreover, it is not easy to directly connect the results of these tests to fundamental biomechanical principles of gait stability, which emphasize the interplay between the movements of the body’s center of mass (CoM) and its base of support (BoS). Herein, we show how a 1.2 m-long treadmill and a single “time-of-flight” Azure Kinect camera can capture the CoM-BoS interplay within 5 min. The CoM was calculated by dividing the body into 14 segments determined from 20 joint positions measured by the Kinect camera’s body tracking SDK. By tracking the CoM and joint positions from stride to stride, we can evaluate different gait stability metrics using a markerless, contactless, space-efficient approach. A large digital database of CoM movements relative to foot placement will be useful for the future development of statistical and machine learning techniques for identifying subjects at higher risk of falling.

## 1. Introduction

Falls are a leading cause of mortality and morbidity in a variety of clinical populations, including the elderly [1], toddlers [2], pregnancy [3], obesity [4], and sensory neuropathies [5]. Ideally, healthcare providers would have access to objective and reliable measures of balance stability during clinic visits, measures that could be used to monitor patient progress and guide timely interventions. Yet, in most outpatient primary care settings, fall risk assessments remain limited to clinical history, subjective questionnaires, and physical maneuvers designed to provoke imbalance [6]. Standard tools such as the Timed-Up-and-Go (TUG) test and the Berg Balance Scales, while convenient, have limited predictive value for fall risk [7]. Although motion analysis technologies (e.g., traditional high-definition multi-camera-based motion capture systems, force plates, and research-grade inertial sensors) are increasingly used in specialized clinics, they remain largely inaccessible in routine care due to high cost, technical complexity, and space requirements [8]. As a result, biomechanical approaches that rely on detailed kinematic measurements, while promising for identifying fall risk, have been mainly confined to motion laboratories and research environments. This inaccessibility limits the feasibility of integrating such assessments into everyday clinical workflows where quick, scalable, and user-friendly tools are essential.

Fortunately, emerging technologies are beginning to close this gap. Unlike traditional systems, markerless motion capture systems (e.g., those using time-of-flight cameras, like the Azure Kinect or Orbbec’s Femto series) [9,10,11] and compact, user-friendly IMUs [12] are specifically designed for portability, ease of use, and low-cost deployment. These newer tools enable high-fidelity motion tracking without the need for markers, wearable suits, or complex lab infrastructure. As such, this offers the potential to bring laboratory-level assessment capabilities into standard outpatient settings [13].

Two key concepts that have been gaining traction in the field of gait stability metrics have been (1) the relationship between the body’s center of mass (CoM) relative to the basin of support (BoS), evaluated by the feet’s placement on the walking surface [14,15,16,17,18] and (2) evaluation of medial–lateral stability, where humans are biomechanically least stable. Regarding the relationship between CoM and BoS, foot placement determines the center of pressure (CoP), the point where corrective forces can be applied to maintain balance. Gait stability requires active coordination between the alternating placements of the feet that affect CoP and the location of the vertical projection of CoM relative to the BoS [14,17]. At the same time, falls in older adults often occur in the medial–lateral direction [1,19,20,21]. Therefore, effective fall risk assessments must include metrics that also capture medial–lateral stability [22]. The challenge has been that measuring both CoM dynamics and foot placement using a single wearable sensor (e.g., an IMU) is not currently possible.

To address this limitation, we propose an alternative approach that leverages treadmill-based gait assessments and computer vision. Although there are slight differences in gait parameters between treadmill and overground walking [23,24], the effect of these differences was outweighed by the practical advantages of reducing the spatial footprint required for gait assessment in the outpatient clinical setting [25]. This approach also has the benefit of eliminating the time needed to attach and sterilize sensors after each use.

In this study, our goal was to demonstrate that a range of gait and stability metrics can be accurately assessed in a clinical outpatient primary care setting using a single Kinect camera and a treadmill. Specifically, we studied our system’s capability to capture the subject’s center of mass (CoM) relative to the subject’s basin of support (BoS), assessing the subject’s medial–lateral stability. We used a treadmill to eliminate the need for clinics to have long stretches of ground for patients to walk across. While we are working towards conducting a large-scale study within the primary care setting on elderly subjects with a high fall risk, we conducted exploratory analyses on healthy individuals to determine whether our approach qualitatively aligns with current findings in the literature. Additionally, one of our participants was later identified as being in early pregnancy and returned for a follow-up session in late pregnancy. Although not part of the original study design, this serendipitous case allowed us to qualitatively evaluate our system’s sensitivity to known gait adaptations during pregnancy as reported in prior literature [26].

## 2. Materials and Methods

The Institutional Review Board of Dell Medical School approved this study. All participants provided their informed written consent before data collection.

### 2.1. Participants

A total of 26 participants (14 women, mean age 29 (range 19–54), 12 men, mean age 31 (range 21–50)) with no musculoskeletal disorders or history of falls were recruited from staff, summer interns, and graduate students working in the Health Discovery Building at Dell Medical School.

### 2.2. Hardware and Software

**Figure 1:**
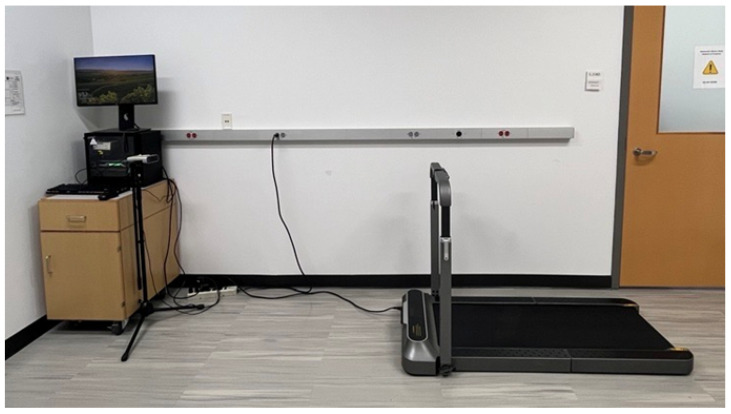
A simple setup with a Kinect camera facing the treadmill and recording computer nearby.

The treadmill walking surface (WalkingPad R2, Beijing Kingsmith Technology, Beijing, China) measured 1.2 m in length and 0.44 m in width. Motion capture was performed using one Azure Kinect sensor (sampling frequency 15 Hz) placed in front of the subject. We found that the camera from the front of the subject was the most consistent in capturing all the joints. A picture of our setup is shown in Figure 1.

The Azure Kinect SDK and Body Tracking SDK packages were installed. Additionally, the latest NVIDIA driver (Nvidia Corporation, Santa Clara, CA, USA) for our graphics card and Visual C++ Redistributable for Visual Studio 2015 (Microsoft, Redmond, WA, USA) were downloaded and installed as part of the Body Tracking SDK software requirements. MATLAB 2022b (MathWorks, Natick, MA, USA) and Python 3/Jupyter Notebook were used for data processing and analysis.

### 2.3. Data Collection

Before data collection began, subjects walked on the treadmill for one minute to familiarize themselves with the device. All subjects self-selected the treadmill speed at which they felt most comfortable. The subjects then walked for about 5 min at their self-selected treadmill speed. We allowed the subjects a minute to find their comfortable walking speed and thus did not start recording until after they had settled. We also did not turn off the treadmill until after 3 minutes of data had been recorded. This decision to record for longer than 3 minutes was made to ensure that we did not capture any artifacts during the on-ramping to or off-ramping from their walking speed. The joint data were aligned so that the medial–lateral (side-to-side) movement ran along the x-axis, the anterior–posterior (forward-backward) movement ran along the y-axis, and the inferior–superior (vertical) movement ran along the z-axis.

### 2.4. Data Processing and Metrics Collection

**Figure 2:**
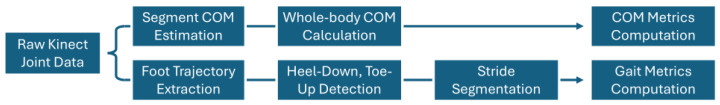
Flow chart of the process to capture both CoM and gait metrics.

Once the data were collected, we extracted features for both gait and center of mass as seen in Figure 2. The list of all the metrics collected is presented in Table 1. The gait metrics we used have been recommended by the Biomathics and Canadian Gait Consortiums Initiative regarding standard gait metrics to capture [27]. The COM metrics we collected are based on studies of fall risk and stability by Tesio et al. [28] as well as Patejak et al. [29]. We compute the ratio between COM and step width as a way to track CoM relative to its BoS, as discussed in a couple of studies [30,31].

#### 2.4.1. Gait Metrics Processing

To track the base of support and calculate various gait metrics, we utilized the foot position recordings. Some joint position data were missing from the Kinect camera, particularly for the feet. If the gap was only one frame, we filled it using linear imputation. If the gap was larger, we excluded that stride from the analysis to avoid misidentifying gait events such as heel-down or toe-up.

**Figure 3:**
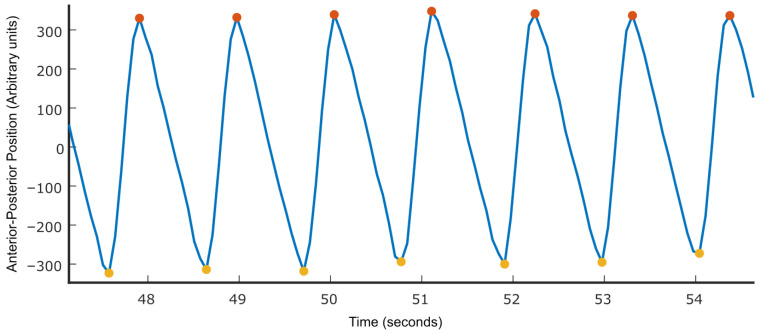
Identification of heel-down (red) and toe-up (yellow) from fore-aft movement of the foot. Because the subject is on the treadmill, the regions with a positive slope represent the movement of the foot above the ground. In contrast, the regions with a negative slope represent the movement of the foot planted on the treadmill.

We were unable to precisely determine the moments of heel contact (“heel-down”) and toe-off (“toe-up”) using a simple threshold on the vertical (*z*-axis) position due to small fluctuations and noise in the joint recordings. To address this, we leveraged motion along the anterior–posterior (*x*-axis) direction, which reflects whether the foot is in contact with the treadmill (moving backward) or in mid-swing (moving forward), as shown in Figure 3. Because participants walked on a treadmill, we could identify gait events based on changes in movement direction. Specifically, heel-down corresponded to the transition from forward to backward motion, and toe-up corresponded to the transition from backward to forward motion. This approach is analogous to the velocity-based method described by Guimarães et al. [32], where the point of minimum heel velocity marks the heel-down event. Although the treadmill introduces continuous backward motion, the foot’s velocity relative to the body still passes through zero during transitions between stance and swing phases. Thus, identifying changes in direction (i.e., zero-crossings) in the anterior–posterior velocity remains a valid indicator of heel-down and toe-up events.

To calculate the gait metrics, we examined the timestamps of when the heel came down, followed immediately by when the toe lifted off. If either of those data points were missing, no time stamp was registered, and that stride calculation would be ignored. The time between the toe lifting off and the heel returning as a single support period by the opposite foot was captured. The average medial–lateral position of the planted foot was stored to help calculate the step width of the strides. A similar process was used to calculate the time when both feet were placed on the floor, using the time between the heel coming down and the opposite foot’s toe lifting off. Through this process, we were able to calculate left stride length, left stride time, right stride length, right stride time, left single support time, right single support time, and double support times for each stride. Because we wanted to track the movement of the CoM relative to the BoS, we also calculated the width of the CoM’s medial–lateral movement as a ratio of the step width.

#### 2.4.2. CoM Metrics Processing

The human body was considered a rigid body system composed of 14 segments: head, neck, chest, upper arms, lower arms, hands, thighs, calves, and feet. The segments were calculated using 20 different joint positions. Based on the position data from the Kinect system, the CoM of each segment was determined separately using

(1) $$P_{i}=P_{u}^{i}-l_{i}*\left(P_{u}^{i}-P_{l}^{i}\right),$$

where $l_{i}$ is the position parameter, $P_{u}^{i}$ is the position of the upper end of the segment, and $P_{l}^{i}$ is the position of the lower end of the segment *i*.

The CoM of the whole body was then calculated by synthesizing the moments using the ratios of the mass of the segments relative to the whole-body mass, $r_{i}$, and the position of the CoM of each segment, $P_{i}$:

(2) $$CoM_{body}=\sum_{i=1}^{N}r_{i}P_{i}$$

Both the position parameters $l_{i}$ and the body mass ratios, $r_{i}$, were obtained from [33].

We examined the CoM trajectory from one left heel down to the next left heel down to capture the behavior of one stride. The width, height, and length of the CoM trajectory were calculated for each stride. To visualize CoM dynamics, we plotted stride-aligned CoM trajectories in each direction and across 2D planes. To align them stride-wise, we normalized the length of each stride by time and plotted the movement of each stride by phase, where 0 and 1 were defined as the boundaries of heel-down to heel-down. Whittle et al. [34] previously observed that the dominant frequency in the medial–lateral direction was twice that of movement in the anterior–posterior and vertical directions. As such, we performed a Fourier analysis on the complete CoM trajectories and compared the dominant frequency in each of these three directions.

### 2.5. Statistical Analysis

For each of the CoM and gait metrics, we conducted two-sample *t*-tests to assess whether there were statistically significant differences between genders. Previous studies have reported that step width is different between male and female [11,35]. Additionally, we fit separate linear regression models to evaluate the influence of walking speed on each metric. Given that we are evaluating across 13 characteristics, we used a Bonferroni-corrected threshold *p*-value of (0.05/13) = 3.85 × 10^−3^ for statistical significance.

### 2.6. Pregnancy Case Study

Unbeknownst to us, one of our subjects was three months pregnant at the time of our initial data recording and was willing to return and re-record her gait patterns at eight months pregnant. A study by Krkeljas showed that due to hormonal and structural changes, pregnancy affects center of mass motion, as gait behaviors change to adapt [26]. In our study, we used data captured from two separate recordings of the same individual. We ran a *t*-test across the three CoM metrics (anterior–posterior, medial–lateral, and vertical movement) to determine whether our setup could also identify these changes.

## 3. Results

### 3.1. Data Quality and Missingness

Given the camera-based nature of our system, some frames were missing due to tracking errors, particularly at the feet. We quantified the extent of the missing data and its impact on stride-level analysis. Across all participants, the average frame loss was 7.9% (SD: 19%), corresponding to an average stride loss of 26.3% (SD: 29%). Upon closer examination, we found that the data from 6 participants had a stride loss greater than 50%. To ensure data quality, we excluded these six participants. Without these participants, the average frame loss dropped to 1.5% (SD: 1.7%), translating to an average stride loss of 12.9% (SD: 13.4%). Most of the gaps extended beyond a single point, and thus, linear imputation was unsuccessful in reducing the frame loss. Regarding CoM calculations, one participant’s head was out of frame, therefore rendering insufficient data for calculating their CoM trajectory.

### 3.2. Statistical Analysis

**Figure 4:**
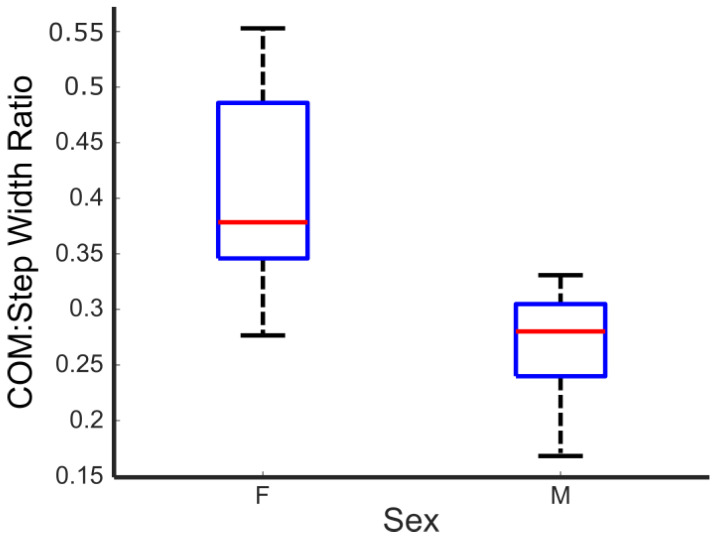
Comparison of CoM/step width ratio by sex. The red line in the box-and-whisker plot represents the median, while the lower and upper bounds of the blue box represents the 25th and 75th percentile respectively.

As seen from Table 2, no gait metrics were statistically significantly different between male and female participants except CoM/step width ratio (Figure 4).

**Figure 5:**
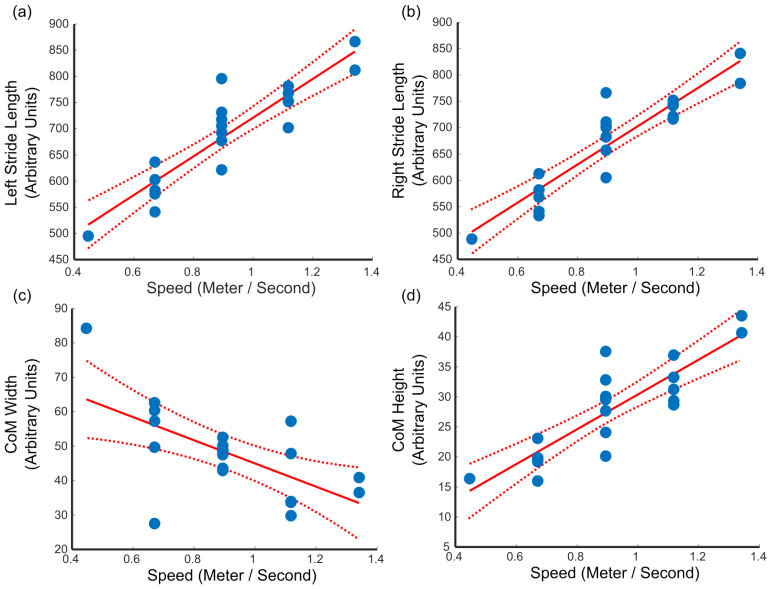
Statistical significant linear relationship between gait speed and (**a**) left stride length, (**b**) right stride length, (**c**) medial lateral range of CoM, and (**d**) vertical range of CoM. Each blue dot represents a single subject. The regression line (solid red) and its 95% confidence interval (dashed red lines) are plotted.

On the other hand, gait speed seems to have strong relationships with specific gait and CoM metrics. We see from Table 3 that gait speed is directly correlated with longer strides, which is expected as longer strides cover more distance. Moreover, there is an associated increase in the amplitude of vertical movement (CoM height) with increased speed (Figure 5) and a decrease in the range of lateral movement (CoM width). This increase in the vertical movements of CoM is not related to an increase in the upper bound of the CoM displacement, but rather to a decrease in the lower bound. The decrease in the lower bound arises because with increased stride length, the CoM lowers. The reduction in lateral movement of the CoM reflects more forward motion than side-to-side motion.

**Figure 6:**
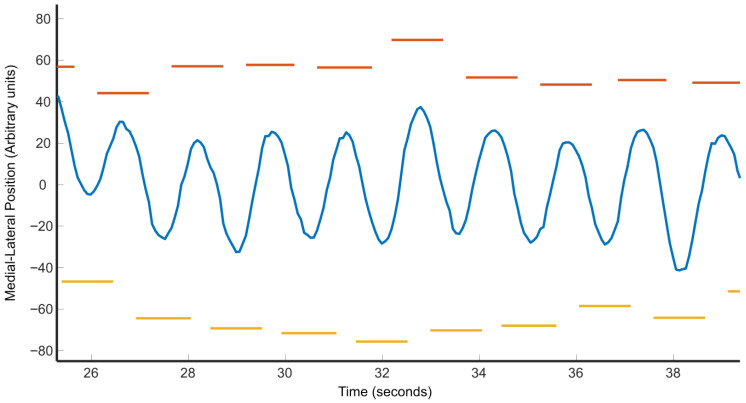
Medial–lateral movement of CoM (blue), with left (orange) and right (yellow) foot placements.

Figure 6 compares the medial–lateral movements of the CoM while walking on the treadmill to step width and the time that each foot is in contact with the surface of the treadmill. As can be seen, when the CoP is located to the right of the CoM (i.e., right foot down), the CoM “falls to the left”. When the CoP is located to the left of the CoM (i.e., left foot down), the CoM “falls to the right” [18]. The relationship between foot placement and medial–lateral movements is of great interest, since gait stability requires that CoM motions be confined within the step width [14,15,16].

**Figure 7:**
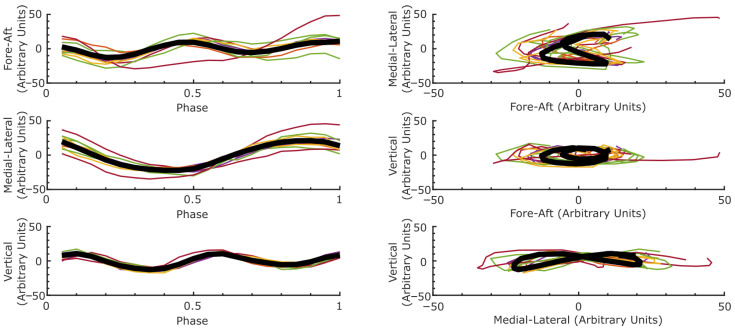
Tracking CoM movements across step phase (**left** column) and across two-dimensional planes (**right** column). The trajectory of each stride is traced in light colors, while the average across the trajectories is plotted in thick black.

The 3D CoM trajectory has been considered the ‘locomotion signature’ of different gaits and speeds [28,36,37]. The movements of the CoM in the anterior–posterior (fore-aft) direction reflect the deceleration at heel strike, followed by the re-acceleration at the push-off and swing phases of each gait cycle. The vertical motions of the CoM provide a measure of the oscillatory energy required to maintain gait posture for a given forward-walking velocity [12]. As is shown in Figure 7, the CoM trajectories exhibit a characteristic “bowtie” or figure-eight shape when plotted in the medial–lateral versus vertical or fore-aft planes, consistent with prior descriptions [28,37,38]. This pattern arises because the medial–lateral CoM oscillations occur at approximately half the frequency of the vertical and fore-aft directions, observed by Whittle et al. [34]. In our data, this is visually evident from the number of oscillatory cycles per stride in each direction. Further, we conducted a frequency analysis of the CoM along each direction. We found the dominant frequency in the medial–lateral direction to be 0.82 Hz compared to 1.63 Hz in the anterior–posterior and vertical directions.

### 3.3. Pregnancy Case Study

The statistical analysis of how CoM changes between 3 and month and 8 month pregnancy is presented in Table 4. As this table shows, there is a statistically significant change in specific CoM metrics. Krkeljas notes specifically the widening of the medial–lateral step width, the lateral trunk lean, and a diminishing of anterior–posterior postural sway [26], all of which can be seen in our data.

**Figure 8:**
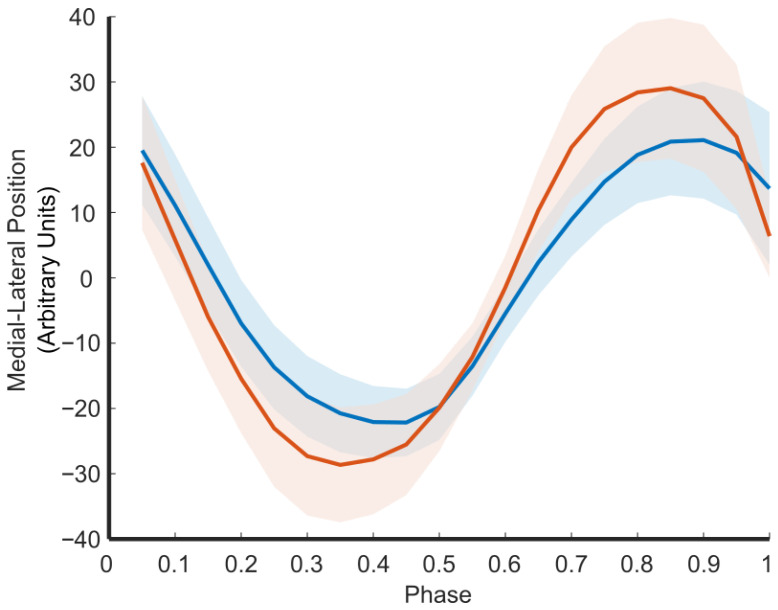
Medial–lateral movement of CoM in one subject at 3 months pregnant (blue) and 8 months pregnant (orange). Mean (line) and one standard deviation are plotted.

Figure 8 shows the medial–lateral changes in gait between these two periods. This figure illustrates how the CoM-BoS interplay changes during pregnancy for a single subject. As can be seen, the width of the CoM is greater at 8 months of pregnancy than at 3 months of pregnancy, reflecting the lateral trunk lean described by Krkeljas [26]. Although we observe that the step width has widened, associated with greater gait stability [39], the ratio between CoM/step width has decreased, which is what we would expect with greater instability.

## 4. Discussions

In the inverted pendulum model of gait, stability is known to depend on both gait speed [40] and the presence of mechanical time delays [41]. Specifically, gait speed influences the condition that the extrapolated center of mass (XCoM) must remain within the BoS. The XCoM extends the CoM by a velocity-dependent factor that decreases with increasing speed. As a result, slower gait speeds allow for a wider margin of stability, making balance more resilient to perturbations. In contrast, mechanical or neural delays (e.g., due to slowed nerve conduction or reduced muscle contraction speed), effectively reduces BoS. For example, in quiet standing, mechanical delays can shrink the effective BoS to nearly one-third of the CoM width [41,42], leading to a higher risk of instability. These observations underscore the destabilizing effects of neuromuscular diseases on gait. Assessing the stability of gait visually during routine physical examinations is inherently challenging. The relationship between the CoM and the center of pressure (CoP) continually changes as gait cycles between the single-leg and double-leg support phases. These inter-relationships become even more challenging to understand in pregnancy and in patients with disorders that affect the musculoskeletal and nervous systems. In this study, we demonstrate that a treadmill combined with a low-cost Kinect camera system enables rapid, quantitative gait stability during a routine clinic visit within 5–10 min.

Before diving into the statistics, one of our initial observations was the instances of missing data in some of our participants. Upon reviewing some of the data, we realized that because this is a camera-based system, if the camera is not positioned properly, with enough margin around the edges to capture the entire skeleton across all phases of the gait, the algorithm will be unable to calculate CoM or gait metrics. Furthermore, we noticed that when participants wore black shoes, the contrast decreased against the black treadmill, resulting in errors if the lighting was not well set. By setting the camera further away to provide more margin and by ensuring proper lighting, these sources of missingness should be easily resolved.

Regarding gait and CoM metrics, our findings revealed statistically significant sex differences in CoM/step width ratio. In contrast, other gait and CoM metrics did not show considerable variation between male and female participants. Interestingly, previous studies have observed that females have narrower step widths than males [35]. This discrepancy may be due to the differences in context between treadmill walking using time-of-flight cameras and overground walking with marker-based tracking systems. Al-Makhalas et al. found statistically significant differences in stride length, with women having shorter stride lengths relative to men [35]. Our study found similar results, but they were not statistically significant in our case. Al-Makhalas et al. noted that this could be due to height differences between males and females, which were also found to be statistically significant.

When examining the relationship between gait speed and gait metrics, we see that gait speed is directly related to stride length and CoM height, while inversely related to CoM width. This finding matches what Baček et al. observed in their recent study [30]: gait speed affecting stride length and not step width. Baček et al. also observed a decrease in lateral CoM movement. They did not make any observations regarding the vertical CoM relationship found in our study. Still, as noted in our results, the longer strides would cause the CoM to dip lower during the double support phase.

In addition to these between-group findings, our pregnancy case study demonstrated the system’s ability to detect meaningful within-subject changes. Between 3 and 8 months of pregnancy, we observed a statistically significant increase in CoM width and vertical displacement, as well as a widening of step width. This increase in CoM width matches what has been observed by Krkeljas [26]. Although step width likely increased to compensate for widening medial–lateral sway, the decreased ratio CoM/step width would suggest insufficient compensation leading to greater instability [30].

While future work is needed to conduct the rigorous validation of our system against more robust motion capture systems, we note that our results qualitatively align with known biomechanical patterns reported in the literature, supporting the value of our time-of-flight-based system for capturing meaningful gait metrics in a clinical setting.

Our system was deliberately designed for clinical feasibility, emphasizing affordability, simplicity, and minimal space requirements. These design choices introduce limitations, particularly in terms of noise and measurement robustness. Future work should explore the trade-offs involved in enhancing system accuracy, such as adding a second camera from a lateral viewpoint. The addition of a second camera could potentially increase measurement noise; thus, any such changes must be carefully weighed against clinical usability constraints.

Despite these limitations, the development of this system opens up the possibility of routine, community-level digital gait assessments. Such data collection could be especially valuable in populations at elevated risk for instability, including pregnant individuals, patients with neurological or orthopedic impairments, and older adults. Routine longitudinal data can help detect subtle changes in gait dynamics before clinical events, such as falls, occur.

In the long term, this type of dataset could enable the application of machine learning to predict fall risk. Gait features (e.g., derived metrics or raw CoM/joint trajectories) can be integrated with traditional clinical tools, such as fall-risk questionnaires and fall history, to train classifiers like random forests, support vector machines, or deep learning models (e.g., LSTMs or transformers). Such predictive systems, deployed in primary care or community health settings, may allow clinicians to shift from reactive to proactive fall risk management, ultimately improving patient outcomes and quality of life.

## Figures and Tables

**Table 1:** Gait and COM metrics collected.

Gait Metrics	CoM Metrics
Stride Length	COM Fore Aft
Stride Times	COM Width
Single Support Times	COM Length
Double Support Times	
Step Width	

**Table 2:** *t*-test analysis of gait metrics by gender. SS represents ‘single support’, while DS represents ‘double support’. Because there were only two categories (male, female), with 19 participants with complete datasets, degrees of freedom (df) was set at 18. Statistically significant metrics are marked with an asterisk.

Metric	Male Mean (SD)	Female Mean (SD)	t-Value	*p*-Value
Left Stride Length	705.39 (111.68)	678.22 (88.83)	0.6069	0.5515
Left Stride Time	0.71 (0.23)	0.74 (0.23)	−0.3605	0.7227
Right Stride Length	689.27 (106.26)	658.91 (87.54)	0.7012	0.4921
Right Stride Time	0.71 (0.24)	0.74 (0.25)	−0.3071	0.7623
Left SS Time	0.58 (0.16)	0.55 (0.25)	0.3062	0.7630
Right SS Time	0.56 (0.17)	0.55 (0.23)	0.0833	0.9345
Left to Right DS Time	0.50 (0.38)	0.46 (0.42)	0.1835	0.8564
Right to Left DS Time	0.48 (0.38)	0.47 (0.41)	0.0861	0.9323
CoM Fore-Aft	31.29 (4.81)	34.17 (5.95)	−1.1704	0.2571
CoM Width	41.56 (10.69)	52.88 (13.09)	−2.0834	0.0517
CoM Height	28.54 (9.66)	27.53 (6.98)	0.2717	0.7889
Step Width	159.79 (37.98)	130.33 (23.95)	2.1159	0.0486
CoM:Step Width Ratio *	0.27 (0.05)	0.41 (0.09)	−4.2519	0.0005

**Table 3:** Linear regression analysis for each gait metric and its relationship with speed. SS represents ‘single stride’, while DS represents ‘double stride’. SE represents ‘Standard Error.’ Statistically significant metrics are marked with an asterisk.

Metric	$\beta_{\mathrm{Speed}}$	SE	t-Value	*p*-Value	$R^{2}$
Left Stride Length *	369.973	41.179	8.985	<0.001	0.818
Left Stride Time	−0.234	0.217	−1.077	0.296	0.061
Right Stride Length *	362.660	37.984	9.548	<0.001	0.835
Right Stride Time	−0.240	0.228	−1.053	0.306	0.058
Left SS Time	−0.091	0.207	−0.441	0.664	0.011
Right SS Time	−0.187	0.192	−0.970	0.345	0.050
Left to Right DS Time	−0.217	0.381	−0.571	0.575	0.018
Right to Left DS Time	−0.240	0.380	−0.633	0.535	0.022
CoM Fore-Aft	−7.951	5.104	−1.558	0.137	0.119
CoM Width *	−33.696	10.162	−3.316	0.004	0.379
CoM Height *	29.022	4.049	7.168	<0.001	0.741
Step Width	10.559	33.076	0.319	0.753	0.006
CoM/Step Width Ratio	−0.253	0.084	−3.023	0.007	0.337

**Table 4:** *t*-test analysis for each CoM metric and its relationship to the month of pregnancy.

Metric	3-Month Mean (SD)	8-Month Mean (SD)	*p*-Value
CoM fore-aft	36.40 (12.51)	33.64 (13.39)	0.0001
CoM width	50.14 (11.18)	58.98 (11.43)	<0.0001
CoM height	27.69 (4.25)	31.60 (3.95)	<0.0001
Step width	138.70 (22.48)	141.67 (22.42)	<0.0001

## Data Availability

The datasets presented in this article are not readily available because the data are part of an ongoing study. Requests to access the datasets and code should be directed to Joshua Chang.
